# Cardiac Clues in Major Depressive Disorder: Evaluating Electrical Risk Score as a Predictive Electrocardiography Biomarker

**DOI:** 10.3390/medicina61061026

**Published:** 2025-05-31

**Authors:** Ulker Atilan Fedai, Halil Fedai, Zulkif Tanriverdi

**Affiliations:** 1 Department of Psychiatry, Faculty of Medicine, Harran University, Sanliurfa 63300, Turkey; 2 Department of Cardiology, Faculty of Medicine, Harran University, Sanliurfa 63300, Turkey; drhalilfedai@gmail.com (H.F.); ztverdi@gmail.com (Z.T.)

**Keywords:** biomarkers, cardiovascular diseases, electrocardiography, electrical risk score, heart rate, major depressive disorder, risk assessment

## Abstract

*Background and Objectives*: Major depressive disorder (MDD) is a prevalent psychiatric illness increasingly recognized as a systemic condition with implications for cardiovascular diseases. Growing evidence indicates that individuals with MDD have an elevated risk of cardiovascular mortality, underscoring the need for reliable, non-invasive biomarkers to assess cardiac risk. While underlying mechanisms remain unclear, electrocardiogram (ECG)-based markers offer a promising, non-invasive means of evaluation. Among these, the electrical risk score (ERS), a composite derived from specific ECG parameters, has emerged as a predictor of adverse cardiac outcomes. This study aimed to investigate the association between ERS and MDD, and whether ERS correlates with depression severity and illness duration. *Materials and Methods*: In this retrospective cross-sectional study, 12-lead ECGs were evaluated to calculate the ERS based on six ECG parameters: heart rate, corrected QT interval, Tp-e interval, frontal QRS-T angle, QRS transition zone, and presence of left ventricular hypertrophy according to Sokolow–Lyon criteria. The Hamilton Depression Rating Scale (HAM-D) was utilized. *Results*: The study included 102 patients with MDD and 62 healthy controls. No significant differences were observed in baseline or laboratory parameters between the groups. However, heart rate, Tp-e interval, frontal QRS-T angle, and ERS were significantly higher in the depression group. ROC analysis identified ERS as the strongest predictor of depression. ERS was significantly higher in patients with severe depression compared to those with mild symptoms and showed a positive correlation with both disease duration and HAM-D score. *Conclusions*: Here, we show that the ECG-derived ERS is significantly elevated in patients with MDD and is associated with increased cardiac risk. ERS outperformed conventional ECG parameters in identifying individuals with depression and demonstrated positive associations with both illness duration and symptom severity. These findings suggest that ERS may serve as a practical, non-invasive biomarker for assessing cardiovascular vulnerability in this population.

## 1. Introduction

Major depressive disorder (MDD) has been identified as the most prevalent mental health condition in the population overall, with a lifetime risk ranging from 6% to 25%. It is projected to be the most significant cause of disability on a global scale by the year 2030 [1,2]. Research has demonstrated that individuals diagnosed with MDD exhibit an elevated risk of developing cardiovascular disease (CVD) [3]. The pathophysiology of MDD includes serotonin reduction, which can lead to excessive sympathetic neural discharge and autonomic nervous system dysregulation [4]. The observed decrease in heart-rate variability and increase in blood pressure in MDD patients may be a result of autonomic nervous system dysfunction [5]. Furthermore, increased blood levels of inflammatory immune markers have been observed in MDD patients [6,7]. In light of these findings, the relationship between autonomic dysfunction and inflammation, and depression and cardiovascular disease may be partially explained [5,7]. A study has demonstrated that individuals diagnosed with CVD exhibit a heightened propensity to develop depression compared to those without CVD. Concurrently, individuals diagnosed with depression are more susceptible to developing CVD compared to those considered to be healthy [8]. A bidirectional relationship between cardiovascular disease (CVD) and depression has been consistently demonstrated in many studies [9,10,11]. It has been demonstrated that both cardiovascular disease and depression are associated with oxidative stress and inflammation [10].

There is evidence that people with severe MDD are more likely to develop CVD [8,12]. An analysis of the studies shows that the two conditions are actually related. In addition, a meta-analysis of several studies demonstrated that depression was correlated with an elevated risk of sudden cardiac death (SCD), ventricular tachycardia/fibrillation, and atrial fibrillation recurrence. This meta-analysis also indicates that individuals diagnosed with depressive disorders have an increased risk of CVD, especially arrhythmias [13].

As demonstrated in previous studies, irregular heart rate variability, reduced baroreceptor sensitivity, and dysregulated parasympathetic and sympathetic activity are the primary mechanisms of arrhythmias [14,15]. Hormonal imbalance, potentially resulting from acute or chronic stress associated with depression, has been demonstrated to cause dysregulation in sympathetic and parasympathetic activity [14,16]. Consequently, the aforementioned dysregulation may be a contributing factor to the heightened cardiovascular mortality observed in individuals grappling with depression.

The electrical risk score (ERS) is a quantifiable parameter that can be measured by means of a 12-lead electrocardiography (ECG). This score encompasses the most significant ECG parameters that have been developed, including heart rate and markers of myocardial depolarization and repolarization [17]. The ERS comprises six fundamental ECG indicators: pulse rate, the presence of LVH, the QRS transition zone, the QTc, the Tp-e, and the frontal QRS-T angle. The aforementioned parameters have been shown to facilitate risk predictions for a variety of diseases [17,18,19,20,21].

Recent evidence suggests that patients with MDD exhibit measurable alterations in cardiac electrophysiology, independent of other medical conditions. Specifically, MDD has been identified as an independent predictor of both the frontal QRS-T angle and the Tp–Te/QT ratio, which are well-established markers of ventricular repolarization heterogeneity and arrhythmic risk. These abnormalities are thought to result from the autonomic nervous system dysregulation commonly observed in MDD, leading to increased susceptibility to malignant ventricular arrhythmias [22]. The ERS incorporates several of these key ECG markers, including fQRST angle and Tp–e interval, enabling a composite, non-invasive assessment of electrocardiographic instability in depressed patients. Therefore, the use of ERS as a primary outcome measure in this study is supported by both mechanistic plausibility and empirical data linking MDD severity to specific electrophysiological changes [22].

The extant literature has demonstrated a relationship between the risk of developing CVD and mortality risk in MDD patients [23,24]. Despite the growing recognition of the bidirectional relationship between MDD and CVD, there remains a significant gap in the literature regarding the integration of these two domains for risk prediction. The identification of risk factors for CVD in MDD patients may be a crucial step in the prevention of CVD. While several studies have explored the cardiovascular risks associated with depression, there is a lack of comprehensive, non-invasive biomarkers that can predict cardiovascular outcomes specifically in MDD patients [25]. The existing research primarily focuses on individual physiological parameters, such as heart rate variability or blood pressure, without considering a holistic, composite measure that accounts for the complexity of both cardiovascular and autonomic dysfunction in depression [25]. The major aim of this study is to investigate the clinical and prognostic significance of the ERS in patients diagnosed with MDD. The primary objective is to ascertain whether ERS, a composite marker derived from standard 12-lead ECG parameters, can serve as a non-invasive tool to predict cardiovascular risk in individuals with MDD. As secondary objectives, we aim to examine the relationship between ERS and both the duration and severity of depressive symptoms, as measured by clinical evaluation. To our knowledge, addressing a significant gap in the literature on cardiovascular risk stratification in depressive disorders, this is the first study to evaluate the utility of ERS in a population with MDD. Moreover, this research contributes to the growing body of evidence advocating for integrative approaches that bridge psychiatry and cardiology, which could ultimately enhance patient outcomes across both disciplines.

## 2. Materials and Methods

### 2.1. Study Design and Data

This retrospective, case–control study was designed to evaluate the utility of the ERS in patients with MDD. The study comprised 102 patients diagnosed with depression; age- and sex-matched 62 healthy controls without any cardiac, psychiatric, and chronic diseases were included in the study. The control group included individuals who were admitted to the psychiatric clinic for non-clinical purposes, such as administrative evaluations or routine check-ups, and were determined to be free of psychiatric disorders based on a structured clinical interview conducted by a psychiatrist in accordance with DSM-5 criteria. Individuals with any current or past psychiatric diagnosis, cardiac disease, or chronic somatic illness were excluded from the control group. The study comprised patients diagnosed with MDD, including both individuals who were currently using antidepressant or psychotropic medications and those who had discontinued treatment at least four weeks prior to ECG assessment. For the medication-free subgroup, the four-week criterion was used to reduce the acute effects of psychotropic drugs on ECG parameters. Medication usage was documented in detail for all patients and is presented in Table 1. This study was conducted among patients seen at the clinic between January 2024 and June 2024. The diagnosis of depression was made according to the DSM-V criteria. Patients over the age of 18 were included in the study. Patients with bundle branch block, paced rhythm, second- or third-degree atrioventricular block on baseline ECG were excluded from the study. The study was conducted according to the Declaration of Helsinki and approved by the Ethics Committee of HarranUniversity.

### 2.2. Electrocardiography

**Figure 1:**
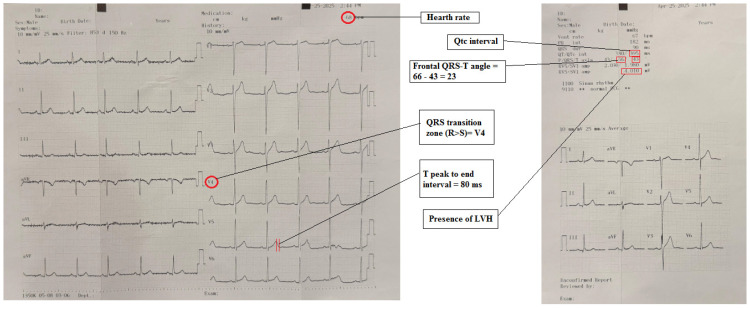
An example of the evaluation of ERS from the 12-lead ECG with its automatic report.

A 12-lead electrocardiogram (ECG) was performed in accordance with standard ECG acquisition rules and following American Heart Association guidelines. The ECGs were evaluated by two independent cardiologists who were unaware of the clinical data. The ERS was calculated using the scores from each ECG. The inter-rater reliability was assessed using Cohen’s kappa coefficient (κ = 0.85), which indicated excellent agreement between the cardiologists. The electrical risk score was then calculated from the ECG (Figure 1). Heart rate, signs of LVH, Tp-e interval, QTc interval, frontal QRS-T angle, and QRS transition zone were used to calculate the ERS. Patients with a heart rate higher than 75/min, presence of LVH according to Skolow–Lyon, QRS transition zone ≥ V4, QTc interval > 450 ms in men and >460 ms in women, Tp-e interval > 89 ms and frontal QRS-T angle > 90° were scored, one point for each criterion [26]. The presence of LVH was determined as the sum of the voltage of the V1S wave + V5/6R wave ≥ 35 mm based on the Sokolow–Lyon criteria [27]. QT interval was calculated from the beginning of the QRS wave to the end of the T wave and corrected using the Bazett formula (QTc Bazett: QT/√RR). The Tp-e interval was calculated as the distance between the peak and the end of the T wave. The QRS transition zone V1-6 precordial leads were analyzed, and the lead in which the R wave was greater than the S wave was identified and recorded [28]. The frontal QRS-T angle was measured as the absolute difference between the QRS axis and the T axis. In instances where this discrepancy exceeded 180°, the angle was calculated by subtracting 360° [29]. As illustrated in Figure 1, an example of ECG interpretation is presented. Scoring was conducted for each parameter. Each parameter was assigned a score, and the total score was determined as ERS (lowest score: 0, highest score: 6) [30].

### 2.3. Hamilton Depression Rating Scale (HAM-D)

The severity of depressive symptoms experienced by participants was evaluated using the Hamilton Depression Rating Scale (HAM-D), a tool that was developed by Max Hamilton [31]. This clinician-administered scale is designed to evaluate the intensity of depressive symptoms experienced during the previous week. The version utilized in this study comprises 17 items, including core domains such as depressed mood, work and activities, somatic symptoms, genital symptoms, weight loss, insomnia (initial, middle, and late), guilt, suicidal ideation, hypochondriasis, somatic anxiety, psychic anxiety, insight, psychomotor retardation, and agitation.

The total score ranges from 0 to 53. Based on the total score, depression severity is classified as follows: 0–7 indicates no depression, 8–15 corresponds to mild depression, 16–28 indicates moderate depression, and scores of 29 or above are considered indicative of severe depression. The Turkish validity and reliability of the HDRS have been established by Akdemir et al. (1996) [32].

### 2.4. Statistical Analysis

The statistical analysis was conducted utilizing the SPSS 22.0 program. The Kolmogorov–Smirnov test was utilized to ascertain the normality of continuous data. Continuous data that conformed to a normal distribution were reported as the mean ± standard deviation and subsequently subjected to Student’s *t*-test for comparison. Continuous data that did not conform to a normal distribution were reported as medians (quartiles 25–75) and analyzed using the Mann–Whitney U test. Categorical variables were expressed as numbers and percentages and compared with the chi-square test. Receiver operating characteristic (ROC) curve analysis was used for QTc interval, Tp-e interval, and frontal QRS-T angle parameters, which were significant or close to significance between the two groups. Pearson or Spearman correlation coefficients were utilized in the context of correlation analysis between the HAM-D scale and ERS. *p* values less than 0.05 were considered to be statistically significant.

## 3. Results

**Figure 2:**
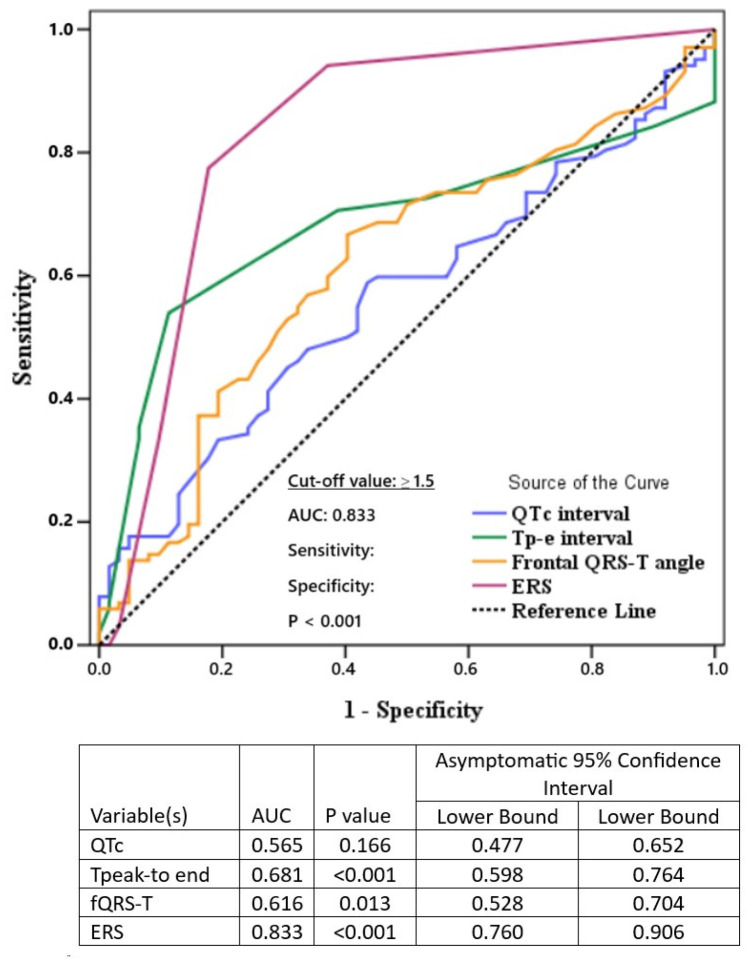
Comparison of the AUC values of the electrocardiographic parameters for predicting the presence of depression.

In our study, which involved 102 patients with depression and 62 control patients, no statistically significant difference in baseline and clinical laboratory parameters was detected between the two groups (Table 1). When ECG parameters were compared between the patient and control groups, Sokolow–Lyon voltage criteria and QTc interval were not statistically significantly different. However, heart rate (83.1 ± 16.3 vs. 70.8 ± 9.5, *p* < 0.001), Tp-e interval (81.3 ± 21.7 vs. 70.1 ± 14.0, *p* < 0.001), and frontal QRS-T angle (28.0 (11.8–53.0) vs. 14.5 (9.0–30.3), *p* = 0.013) were higher in the patient group and this increase was statistically significant (Table 2). In addition, ERS calculated from ECG parameters was statistically significantly higher in the patient group than in the control group (0 (0–1) vs. 2 (2–3), *p* < 0.001). Furthermore, ROC curve analysis with QTc interval, frontal QRS-T angle, Tp-e interval, and ERS showed that ERS was the best predictor of depression with the best AUC value (area under curve = 0.833, 95% confidence interval: 0.760–0.906, *p* < 0.001) (Figure 2). The optimal cut-off value for ERS was calculated as **1.5**, which yielded a sensitivity of **77.5%** and a specificity of **82.3%**.

**Figure 3:**
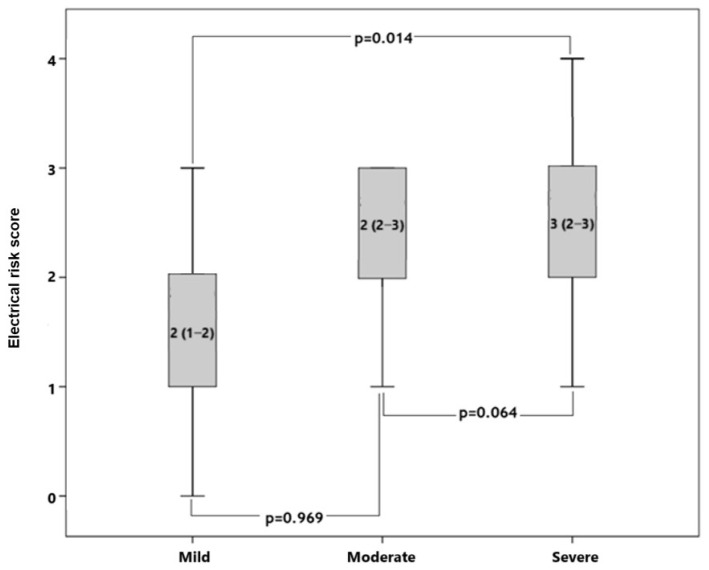
Comparison of ERSs among the patients with mild, moderate, and severe HAM-D scores.

**Figure 4:**
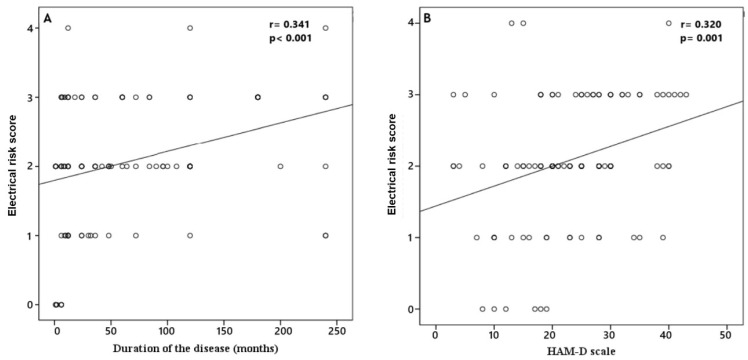
Correlation analysis of ERS with duration of the disease (**A**) and HAM-D scale (**B**). Circles indicate data points, and lines represent the correlation line.

In the patients’ group, the median duration of the disease was 36 (12–120) months, and the HAM-D was 23 (17–30). Our patient group was divided into three groups according to the HAM-D scale as follows: mild (score between 8 and 15), moderate (score between 16 and 28), and severe (score ≥ 29). It was observed that ERS was significantly different among the three groups (*p* = 0.012). In subgroup analyses, we found that patients with severe HAM-D scores had significantly higher ERSs compared to patients with mild HAM-D scores (Figure 3). In addition, correlation analysis demonstrated that ERS was positively correlated with duration of the disease (r = 0.341, *p* < 0.001) and HAM-D scale (r = 0.320, *p* = 0.001) (Figure 4).

## 4. Discussion

This study investigates the prognostic and clinical significance of ERS in patients with MDD. The main results of our study are as follows: heart rate, Tp-e interval, and frontal QRS-T angle and ERS were significantly greater in the patient group compared to the control group. ROC analysis of ERS, heart rate, Tp-e interval, and frontal QRS-T angle showed that ERS had a better AUC value than other ECG parameters in the patient group. Furthermore, a positive correlation was found between ERS and both disease duration and severity. To our knowledge, this is the first research to investigate the ERS in patients with MDD and makes a novel contribution to the interface between psychiatry and cardiology.

It is important to note that MDD has the potential to evolve into a chronic condition, characterized by recurrent attacks. This progression may also be accompanied by the development of comorbid diseases [33]. MDD has been established as a significant risk factor for cardiovascular disease [34,35]. It is hypothesized that one of the factors causing this relationship is autonomic nervous system (ANS) dysregulation [14]. It is thought that decreased serotonin levels in major depression may lead to overstimulation of sympathetic neural discharge [14]. Decreased parasympathetic and increased sympathetic activity may lead to myocardial ischaemia, ventricular tachycardia, ventricular fibrillation, and sudden cardiac death [36]. In conclusion, it can be posited that dysregulation of the ANS caused by major depression may lead to alteration of cardiac autonomic tone and initiation of procoagulant and proinflammatory processes [36]. Another hypothesis is that emotional stress can cause inflammation and cortisol release [37]. Many studies have shown that long-term stress-induced inflammation and cortisol exposure may lead to vascular deterioration and atherosclerosis [36,38]. In addition to these points, it has also been shown that sensory stress has a significant effect on platelet activation [39]. This suggests that major depression may lead to an elevated incidence of coronary heart disease (CHD) and sudden cardiac events [36].

Although the examination of 12-lead ECG is a simple and widely accessible diagnostic tool, it remains a crucial component in the evaluation of cardiovascular disease and mortality risk [40]. A review of the current literature reveals numerous studies employing various ECG parameters to explore the association between MDD and cardiac arrhythmias, dysregulated cardiac autonomic activity, CHD, and cardiac mortality [14,22]. More recently, the ERS has emerged as a valuable tool for assessing cardiac risk. ERS is a composite risk score that incorporates multiple ECG parameters to provide insights into potential cardiac events [17]. It has been proposed as a predictor of sudden cardiac death. This score evaluates parameters such as heart rate, QTc, and Tp-e, reflecting imbalances in neuro-autonomic regulation. Additionally, it captures repolarization abnormalities through QTc, QRS angle, and Tp-e measurements, and indicates cardiac hypertrophy through QTc, QRS angle, QRS transition, and Tp-e parameters. Given its comprehensive nature, ERS has been identified in several studies as a reliable predictor of cardiac mortality [17,18]. A review of the literature on ERS revealed few studies. Initially, a correlation between ERS and sudden cardiac death was identified in the high cardiovascular risk group [17]. A study of patients undergoing transcatheter aortic valve replacement and a study of non-ST-elevation myocardial infarction patients demonstrated that ERS is a robust predictor of all-cause mortality [18,41].

Our findings demonstrated that Tp-e interval and frontal QRS-T angle (markers of myocardial depolarization and repolarization) were significantly elevated in patients with MDD versus healthy controls. It has been documented that an augmentation in these parameters is related to an increased risk of ventricular arrhythmia [42]. It has been established that an imbalance in the ANS is the most common factor contributing to ventricular repolarization heterogeneity [22]. Prior studies have demonstrated that there is ANS dysregulation in MDD [43]. In another study conducted with depressed patients, similar to the present study, fQRS-T angle and Tp-e interval were observed to be significantly higher [17]. The analysis of heart rate and Tp-e interval indicates an imbalance in neuro-autonomic control [17,18]. It has been established that there is an increase in sympathetic discharge and a decrease in parasympathetic discharge in individuals diagnosed with major depression [36]. Changes in these parameters can be considered as evidence of this imbalance. In the present study, heart rate and Tp-e interval were observed to be significantly higher in patients with depression versus to the control group.

The RoC analysis was performed to compare Qtc, Tp-e, fQRS-T angle, and ERS. Our findings suggest that ERS is a superior indicator of mortality and morbidity in depression cases when compared to other ECG parameters. A review of studies conducted on patients diagnosed with major depression reveals significant variations in fQRS-T angle, Tp-e interval, and Qtc interval [14,22]. However, in the present study, all of these parameters were evaluated cumulatively, and ERS emerged as a superior indicator in the ROC analysis. With this finding, our study reveals a significant difference from other studies.

A positive correlation was identified between the duration of the disease and the severity of the disease and ERS. Consequently, as the duration of the disease increases, so too does the risk of cardiac morbidity and mortality. Furthermore, a significant difference in ERS severity was found when comparing mild and severe depression cases. ERS has been demonstrated to be elevated in individuals afflicted with severe depression, and there is a concomitant increase in cardiac morbidity and mortality. Similarly, a study reported a positive correlation between the severity of depression and fQRS-T angle and Tp-e/QTc ratio and found an increased risk of arrhythmia [22]. This study provides more comprehensive information compared to previous research. Therefore, it has the potential to offer more valuable insights regarding cardiac mortality.

The present study is not without its limitations. The most significant limitation is the retrospective design of the study and the comparatively lower number of patients. A further potential limitation is that some patients received antidepressant treatment. While individuals classified as “medication-free” had discontinued pharmacologic therapy at least four weeks prior to ECG acquisition to minimize acute drug effects, a portion of the sample was receiving ongoing treatment at the time of assessment. However, it should be noted that the antidepressants administered to our patients were predominantly serotonin-noradrenaline reuptake inhibitors and selective serotonin reuptake inhibitors, which have been known to exhibit a reduced propensity for cardiotoxicity when compared to tricyclic and monoamine antidepressants [44]. Nevertheless, we acknowledge that residual or long-term physiological effects of past or ongoing pharmacologic treatments on ECG parameters (including ERS) cannot be entirely excluded and may represent a source of confounding. Another limitation of our study is the lack of data on the presence of anxiety and comorbid somatic symptoms. These factors may independently influence cardiovascular autonomic markers and could act as potential confounding variables. Future studies should consider including these variables to better understand their impact on the findings. Due to the cross-sectional nature of the study, the relationship between the variables cannot be clearly demonstrated. Therefore, a prospective or interventional follow-up study is necessary to validate the predictive utility of ERS in MDD over time, which will provide a more in-depth temporal perspective.

In summary, this study highlights the utility of the ERS as a promising, non-invasive tool for identifying elevated cardiac risk in patients with MDD. A major strength of the study is its novel integration of multiple ECG parameters into a composite score, allowing for a more holistic assessment of cardiovascular risk compared to isolated markers. Despite the limitations of its retrospective design and modest sample size, the study offers valuable preliminary insights into a clinically relevant population. If validated in future prospective, longitudinal research, the ERS could aid in stratifying cardiovascular risk in psychiatric populations, contributing to early intervention strategies and interdisciplinary care models. These findings bridge an important gap between cardiology and psychiatry and may inform future risk assessment protocols in mental health settings.

## 5. Conclusions

In conclusion, the present study provides novel insights into the prognostic and clinical significance of the ECG-derived ERS in patients diagnosed with MDD. The findings indicate that ERS, along with associated parameters such as heart rate, Tp-e interval, and frontal QRS-T angle, are significantly elevated in individuals with MDD, suggesting an increased risk of cardiac morbidity and mortality. Importantly, ERS demonstrated superior predictive value in predicting mortality and morbidity risk in MDD patients compared to traditional ECG markers, as evidenced by ROC analysis. The positive correlation between ERS and both disease duration and severity further highlights the usefulness of ERS as a non-invasive biomarker for assessing cardiovascular risk in this patient population. Our findings add to the growing body of literature that highlights the connection between mental and cardiovascular health. They also emphasize the importance of incorporating cardiac risk assessment into the routine clinical evaluation of individuals with depression. From a clinical perspective, these results advocate for early identification and cardiovascular monitoring in patients with moderate to severe depression, potentially guiding risk stratification and preventative strategies. Despite the study’s retrospective nature and limited sample size, these results highlight the importance of cardiac monitoring in individuals with MDD and emphasize the need for prospective studies with larger cohorts to validate and expand upon these findings.

## Figures and Tables

**Table 1:** Comparison of baseline clinical and laboratory markers of study groups.

Variables	Patients (*n* = 102)	Control (*n* = 62)	*p*
Age, years	39.0 (28.8–51.3)	35.5 (27.0–48.3)	0.465
Gender, female (%)	62 (60.8)	36 (58.1)	0.731
Smoking (%)	34 (33.3)	28 (45.2)	0.130
SBP, mmHg	119.9 ± 7.7	120.9 ± 8.3	0.425
DBP, mmHg	72.0 ± 6.6	71.1 ± 6.3	0.375
Creatinine, mg/dL	0.79 ± 0.18	0.81 ± 0.19	0.449
Total-cholesterol, mg/dl	219.1 ± 48.7	220.8 ± 53.7	0.829
Triglyceride, mg/dL	134.5 (83.3–199.5)	126.5 (77.8–168.0)	0.391
LDL-cholesterol, mg/dL	137.2 ± 38.7	135.0 ± 39.4	0.735
HDL-cholesterol, mg/dL	53.6 ± 13.3	56.2 ± 14.5	0.251
Hemoglobin, g/dL	13.9 ± 1.7	13.4 ± 1.6	0.067
Platelets, ×10^3^/µL	269.2 ± 63.3	267.2 ± 67.9	0.853
Leukocytes, ×10^3^/µL	7.6 ± 1.6	7.2 ± 1.9	0.103
TSH, mIU/L	1.9 (1.2–2.5)	1.8 (1.3–2.5)	0.948
Medication (%)	55 (53.9)		
	**n (min–max mg/day)**		
Venlafaxine	34 (75–375)		
Duloxetine	2 (60–120)		
Sertraline	14 (50–200)		
Essitaloprame	1 (10)		
Fluoxetine	4 (40)		
Paroxetine	3 (20–40)		
Mirtazapine	18 (15–45)		
Trazodone	4 (50–100)		
Bupropione	7 (150–300)		
Klomipramine	1 (75)		
Olanzapine	12 (2.5–10)		
Quetipine	16 (25–300)		
Aripiprazole	2 (2.5–5)		
Sulpırıde	4 (50–100)		

SBP: systolic blood pressure, DBP: diastolic blood pressure, TSH: thyroid-stimulating hormone.

**Table 2:** Comparison of electrocardiographic markers of study groups.

	Patients (*n* = 102)	Control (*n* = 62)	*p*
Heart rate, /bpm	83.1 ± 16.3	70.8 ± 9.5	<0.001
Sokolow–Lyon voltage criteria, mV	17.8 (12.9–21.9)	18.0 (15.0–22.3)	0.205
QTc interval, ms	414.8 ± 29.0	408.3 ± 18.4	0.081
Tp-e interval, ms	81.3 ± 21.7	70.1 ± 14.0	<0.001
Frontal QRS-T angle, ^o^	28.0 (11.8–53.0)	14.5 (9.0–30.3)	0.013
ERS	2 (2–3)	0 (0–1)	<0.001

ERS: Electrocardiographic Risk Score.

## Data Availability

The data presented in this study are available upon request from the corresponding author. The data are not publicly available due to the arrangements made by the Ethics Committee.

## Abbreviations

The following abbreviations are used in this manuscript:

MDD	Major depressive disorder
CVD	Cardiovascular disease
SCD	Sudden cardiac death
ERS	Electrical risk score
ECG	Electrocardiography
HAM-D	Hamilton Depression Rating Scale
ROC	Receiver operating characteristic
ANS	Autonomic nervous system
CHD	Coronary heart disease
