# Correction: Atilan Fedai et al. Cardiac Clues in Major Depressive Disorder: Evaluating Electrical Risk Score as a Predictive Electrocardiography Biomarker. *Medicina* 2025, *61*, 1026

**DOI:** 10.3390/medicina61071203

**Published:** 2025-07-01

**Authors:** Ulker Atilan Fedai, Halil Fedai, Zulkif Tanriverdi

**Affiliations:** 1 Department of Psychiatry, Faculty of Medicine, Harran University, Sanliurfa 63300, Turkey; 2 Department of Cardiology, Faculty of Medicine, Harran University, Sanliurfa 63300, Turkey; drhalilfedai@gmail.com (H.F.); ztverdi@gmail.com (Z.T.)

**Error in Table**

In the original publication, there was a mistake in **Table 2** as published [1]. **The authors could not notice this error in the proof file.** The corrected **Table 2** appears below. The authors state that the scientific conclusions are unaffected. This correction was approved by the Academic Editor. The original publication has also been updated.

## Figures and Tables

**Table 2:** Comparison of electrocardiographic markers of study groups.

	Patients (*n* = 102)	Control (*n* = 62)	*p*
Heart rate, /bpm	83.1 ± 16.3	70.8 ± 9.5	<0.001
Sokolow–Lyon voltage criteria, mV	17.8 (12.9–21.9)	18.0 (15.0–22.3)	0.205
QTc interval, ms	414.8 ± 29.0	408.3 ± 18.4	0.081
Tp-e interval, ms	81.3 ± 21.7	70.1 ± 14.0	<0.001
Frontal QRS-T angle, ^o^	28.0 (11.8–53.0)	14.5 (9.0–30.3)	0.013
ERS	2 (2–3)	0 (0–1)	<0.001

ERS: Electrocardiographic Risk Score.

## References

[1] Atilan Fedai U., Fedai H., Tanriverdi Z. (2025). Cardiac Clues in Major Depressive Disorder: Evaluating Electrical Risk Score as a Predictive Electrocardiography Biomarker. Medicina.

